# Throat-Ail Causes

**Published:** 1877-01

**Authors:** 


### THROAT AIL CAUSES.

A RESPECTED correspondent writes : "I took cold three weeks ago, preaching in a draft. It affected my throat and ears also, so that in speaking I was annoyed by a jingling kind of sound. I was foolish in taking it as I did." We replied in part, "Preaching in the draft has destroyed many a valuable clergyman. You should make up your mind never to do it again. Stand in the corner of the room. Is it likely that the effect of any given sermon is worth a minister's life, when he otherwise might have lived twenty years longer, with the chances of repeating this effect thousands of times? Why don't you preachers study ecclesiastical economy more?"

We must say that clergymen many times act as if they were made out of steel, as if their health was impregnable, and that nothing could effect it injuriously. They ought to remember that they have no charmed lives, and that their only secure shield against the shafts of disease and death, which fly around all at every step, is a RATIONAL CARE.

